# A Method to Determination of Lead Ions in Aqueous Samples: Ultrasound-Assisted Dispersive Liquid-Liquid Microextraction Method Based on Solidification of Floating Organic Drop and Back-Extraction Followed by FAAS

**DOI:** 10.1155/2018/8951028

**Published:** 2018-08-01

**Authors:** Çiğdem Arpa, Itır Aridaşir

**Affiliations:** Chemistry Department, Hacettepe University, Beytepe, 06800 Ankara, Turkey

## Abstract

Ultrasound-assisted dispersive liquid-liquid microextraction method based on solidification of floating organic drop and back-extraction (UA-DLLME-SFO-BE) technique was proposed for preconcentration of lead ions. In this technique, two SFODME steps are applied in sequence. The classical SFODME was applied as the first step and then the second (back-extraction) step was applied. For the classical SFODME, Pb ions were complexed with Congo red at pH 10.0 and then extracted into 1-dodecanol. After this stage, a second extraction step was performed instead of direct determination of the analyte ion in the classical method. For this purpose, the organic phase containing the extracted analyte ions is treated with 1.0 mol·L^−1^ HNO_3_ solution and then exposed to ultrasonication. So, the analyte ions were back-extracted into the aqueous phase. Finally, the analyte ions in the aqueous phase were determined by FAAS directly. Owing to the second extraction step, a clogging problem caused by 1-dodecanol during FAAS determination was avoided. Some parameters which affect the extraction efficiency such as pH, volume of extraction solvent, concentration of complexing agent, type, volume, and concentration of back-extraction solvent, effect of cationic surfactant addition, effect of temperature, and so on were examined. Performed experiments showed that optimum pH was 10.0, 1-dodecanol extraction solvent volume was 75 *μ*L, back-extraction solvent was 500 *μ*L, 1.0 mol·L^−1^ HNO_3_, extraction time was 4 min, and extraction temperature was 40°C. Under optimum conditions, the enhancement factor, limit of detection, limit of quantification, and relative standard deviation were calculated as 81, 1.9 *μ*g·L^−1^, 6.4 *μ*g·L^−1^, and 3.4% (for 25 *μ*g·L^−1^ Pb^2+^), respectively.

## 1. Introduction

Lead is highly toxic and potentially dangerous to living organisms because of the accumulation ability in the vital organs. Long-term exposure to lead causes cumulative poisoning which leads to central nervous system damages [1, 2]. On account of this, even at low concentration, determination and controlling of lead content are crucial [3]. However, because of their complex matrix and trace concentration, determination of heavy metals in many real samples is a difficult analytical task and requires sensitive detection techniques and probably a preconcentration procedure [4].

Nowadays, for determination of trace heavy metals, atomic absorption spectrometry equipped with flame (FAAS) or graphite furnace (ETAAS) and inductively coupled plasma emission spectrometry (ICP) [5–7] are widely used analytical techniques. Among these techniques, although flame atomic absorption spectrometry is commonly used for heavy metal determination, its sensitivity is not enough to direct determination of trace and ultra-trace amount of heavy metals in real samples; therefore, some pretreatment procedures, such as separation and preconcentration, are needed [8]. Several sample preparation methods for the preconcentration and separation of heavy metals have been developed. Solid-phase extraction (SPE) [9], solid-phase microextraction (SPME) [10], dispersive liquid-liquid microextraction (DLLME) [11], and cloud point extraction (CPE) [12, 13] are some of these methods [14].

Recently, the solidified floating organic drop microextraction (SFODME) [15–17] has been attracted more attention and properly utilized as an effective method for heavy metal extraction and preconcentration. In this technique, a free microdrop of the organic solvent which has a low melting point (in the range of 10–30°C) and a density lower than water is gently dropped to the stirred sample solution. Under the appropriate stirring conditions, the organic microdrop floats on the aqueous sample and species, which have a hydrophobic character, are extracted into this phase. After completion of the extraction, the sample vial is placed in an ice bath, and the floated drop solidifies. After solidification, the organic drop is easily transferred to a conical tube and melted there. Finally, the analyte in the extraction phase is determined by using FAAS. The SFODME method needs very small volumes of the harmful organic solvent, and this makes the method simple, fast, cheap, and environmental friendly [18]. Application of ultrasonic energy to a solution causes acoustic cavitation. Cavitation is the formation and then immediate implosion of bubbles in a liquid. When high temperatures and pressures are produced at the interface of the bubble and other phases, an increase in chemical reactivity occurs [19]. Hence, combination of microextraction processes with ultrasound energy accelerates the extraction step and improves the efficiency of preconcentration. This combined technique is called as ultrasound-assisted emulsification-microextraction (USAEME) and bears all the advantages of both methods.

Besides all these advantages mentioned above, SFODME has some important disadvantages: selected extracting solvent should not be volatile and toxic, and its melting point should be close to or below room temperature [20]. Having a melting point near room temperature also means that the extracting solvent freezes easily at room temperature. After solidification, the microdrop is transferred to a conical vial, is diluted with an appropriate solvent to a certain volume, and is then analyzed for determination of heavy metal concentration. But, unfortunately, after detection of the analyte, the solvent residues freeze again in the capillaries, tubes, and pipes of the FAAS instrument, and this causes clogging of the sample introduction path and drainage path of the instrument. (Especially, when the room temperature is slightly below 24°C in winter). Remedy of clogging in the system requires washing the pipes with plenty of hot water and alcohol solution several times, which is a time- and chemical-consuming process. In addition to clogging problems, 1-dodecanol has a background signal, which interferes with the analyte signal. To eliminate this problem, the back-extraction step can be applied [1].

In this ultrasound-assisted dispersive liquid-liquid microextraction method based on solidification of floating organic drop and back-extraction (UA-DLLME-SFO-BE) technique, two solidified floating organic drop microextraction (SFODME) steps are performed in sequence. In the first step, the following conventional SFODME procedure is applied: the extraction solvent is added into the solution including heavy metal ions that form hydrophobic complexes with suitable complexing agent. After applying ultrasound energy, extraction occurs, and hydrophobic analyte species are transferred into the organic solvent. At this stage, instead of direct analysis of the extraction phase, a second SFODME round is performed, in which the extraction phase is treated with diluted acid solution, and after applying ultrasound, interested analyte is back-extracted into the aqueous phase. At the end of second SFODME, the obtained aqueous solution, which includes the analyte, is injected to the FAAS. The second extraction step provides the elimination of the clogging problem of the organic extraction solvent.

**Figure 1:**
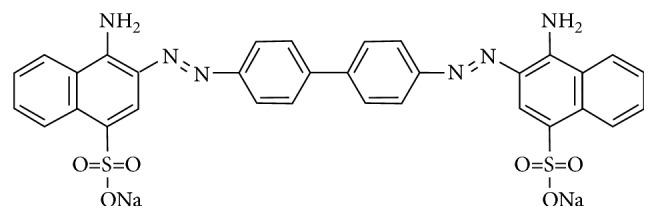
Molecular structure of CR.

In this study, we developed a UA-DLLME-SFO-BE method for the determination of Pb^2+^ ions in aqueous samples. For this purpose, Congo red (CR) is used as a complexing agent to obtain hydrophobic Pb^2+^ complexes. CR-Pb^2+^ complexes are extracted into 1-dodecanol and back-extracted into aqueous nitric acid solution, which eliminates the drawbacks of the organic extraction phase as mentioned above. CR (3,3′-[[1,1′ biphenyl]-4,4′-diylbis-(azo)] bis[4-amino-1-naphthalene-sulfonic acid] disodium salt) is a sulfonated bis-azo dye [21]. As seen from Figure 1, CR has azo, sulfonate, and amino groups, and these groups provide two different donor atoms: one is N from azo or amino groups and other one is O from sulfonate groups. Complexation between metals and CR may occur through these donor atoms [22, 23].

There are two special features at the heart of the study: (i) implementation of ultrasound energy shortens the extraction time and improves the extraction efficiency; (ii) by performing a second extraction step, drawbacks of 1-dodecanol in conventional SFODME (clogging of pipes and tubings of instrument and background signal) are eliminated. In order to fully characterize the proposed method, several parameters were explored and optimized.

## 2. Experimental

### 2.1. Reagents and Standard Solutions

1000 mg·L^−1^ stock standard solution of lead was prepared by dissolving a necessary mass of Pb(NO_3_)_2_ in a small amount of deionized water and diluting it to the appropriate volume. All solutions were prepared with deionized water (18.1 MΩm^−1^) obtained from a Millipore Simplicity UV purification system. Working standard solutions were prepared daily by serial dilutions of the stock solution with deionized water just before analysis. The chelating agent, 0.1% CR solution, was prepared by dissolving the appropriate amount of CR (purchased from BDH, Poole, England) in acetone. Na_2_HPO_4_/NaOH (purchased from Merck, Darmstadt, Germany) buffer system was used to adjust the sample pH to 10.0. 1-dodecanol, used as an extraction solvent, was obtained from Merck, and didecyldimethyl ammoniumchloride (DDMAC) was purchased from Sigma-Aldrich. A solution of 8% (v/v) DDMAC was prepared by dissolving proper amount of DDMAC in water. All reagents used were of analytical reagent grade. All the solutions were prepared by using deionized water. In order to eliminate possible contamination, all glassware was immersed into 10% hydrochloric acid for at least 24 h and then rinsed three times with deionized water.

### 2.2. Instrument

A Perkin Elmer Analyst atomic absorption spectrometer equipped with a lead hollow cathode lamp (Perkin Elmer) as the radiation source was used for determination of lead. The analytical wavelength (283.3 nm), slit width (0.7 nm), and lamp current (28 mA) were used as recommended by the manufacturer. All pH measurements were carried out with an Isolab Laborgeräte GmbH digital pH meter equipped with a pH electrode. A Hettich Eba 21 model centrifuge was used to accelerate the phase separation. A 53 kHz, 100 W temperature-controlled ultrasonic bath (Kudos SK3310LHC) was used in the ultrasound-assisted emulsification process of the method.

### 2.3. Procedure for UA-DLLME-SFO-BE

**Figure 2:**
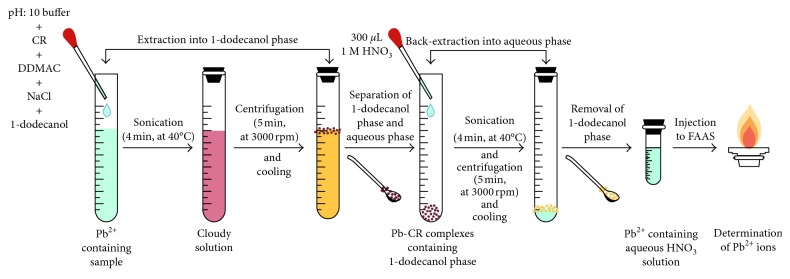
Schematic representation of UA-DLLME-SFO-BE procedure.

The UA-DLLME-SFO-BE method includes two SFODME steps. In the first step, 25.0 mL Pb^2+^ solution or water samples, 2 mL of Na_2_HPO_4_/NaOH buffer solution, 1 mL of 0.5% (w/v) CR solution, 100 *μ*L 8% (v/v) DDMAC, and 0.1 g NaCl were mixed. Then, 75 *μ*L of 1-dodecanol was added. The conical tube was sonicated for 4 min at 40°C to ensure complete extraction. At this step, the lead ions reacted with CR and extracted into 1-dodecanol. With the addition of DDMAC, Pb-CR complex gained more hydrophobic character and was more extractable into the 1-dodecanol phase. With the aid of centrifugation (5 min at 3000 rpm), very small droplets of the 1-dodecanol group were brought together and collected at the vicinity of sample solution. After cooling of the test tube in the refrigerator, the 1-dodecanol phase was solidified, and this solidified phase was transferred into another conical tube. At this stage, instead of adding a diluent and determination of Pb^2+^, a second SFODME procedure was applied. For this purpose, the 1-dodecanol phase containing Pb-CR complexes was treated with 500 *μ*L of 1 mol·L^−1^ HNO_3_ solution. After sonication for 4 min at 40°C, the tube was centrifuged for 5 min at 3000 rpm. At this stage, Pb^2+^ ions were transferred from the 1-dodecanol phase to the aqueous HNO_3_ phase. Finally, the supernatant was introduced into FAAS by direct nebulization for Pb^2+^ analysis, and the blank was also treated in the same way. Schematic representation of the proposed procedure is shown in Figure 2.

## 3. Results and Discussion

In order to obtain the maximum extraction efficiency and the highest enhancement factor, several parameters that influence the metal-ligand formation and the extraction conditions were studied. In the course of optimization experiments, 100 *μ*g·L^−1^ of Pb^2+^ working solutions were utilized. After obtaining optimum conditions, the Pb^2+^ content of certified reference materials and some natural water samples were determined by using these optimum conditions.

### 3.1. Selection of the Extraction Solvent and Effect of Its Volume on Extraction

In the optimization of the SFODME process, the choice of the proper extraction solvent is one of the important steps. For this method, the extraction solvent must have the following features: (1) being immiscible with water, (2) having low volatility, which provides a stable character during the extraction step, (3) having a considerable extraction efficiency, (4) having a lower density than that of water, (5) having a melting point around room temperature (in the range of 10–30°C), and (6) having a low toxicity level [24]. For this purpose, 1-dodecanol (density: 0.8201–0.8309 g·mL^−1^; melting point: 24°C) was chosen as the extracting solvent because of its sensitivity, stability, low water solubility, low vapor pressure, and low price.

To investigate the effect of the extraction solvent volume on extraction efficiency, 1-dodecanol volumes were changed in the range of 10–250 *μ*L, while other parameters were kept at optimum values. It was not possible to work with volumes less than 10 *μ*L. The extraction efficiency values (calculated from absorbance values obtained various volumes of 1-dodecanol) showed that initially increasing the volume of 1-dodecanol in the range of 10–50 *μ*L causes increase in the extraction efficiency, and then the extraction efficiency remained constant at 98% in the range of 75–100 *μ*L. Finally, extraction efficiencies slightly decreased by the volumes greater than 100 *μ*L. Therefore, in the subsequent studies, 75 *μ*L was chosen as the optimum volume for 1-dodecanol.

### 3.2. Selection and Effect of Back-Extraction Solvent

**Figure 3:**
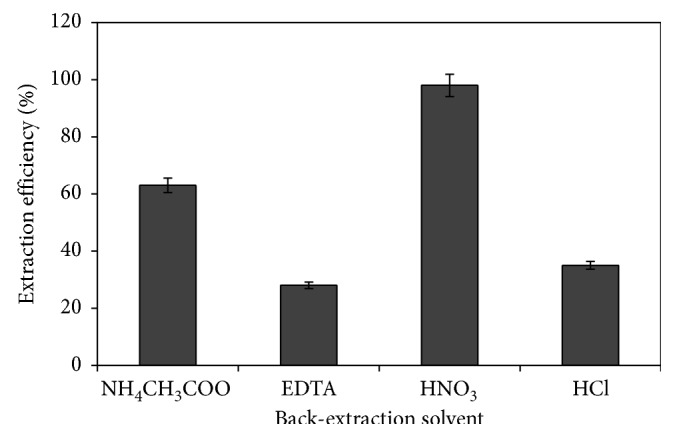
Effect of back-extraction solvent type. Conditions: 25 mL of 100 *μ*g·L^−1^ Pb^2+^, 0.5% (w/v) CR, 0.03% (v/v) DDMAC, 0.1 g NaCl, 75 *μ*L 1-dodecanol, 4 min extraction time, and 40°C extraction temperature.

In the second step of the procedure, several back-extraction solvents were tried to extract Pb^2+^ ions from their Pb-CR complexes into the aqueous phase in order to eliminate drawbacks of the 1-dodecanol phase. For this purpose, aqueous solutions of EDTA, NH_4_CH_3_COO, HCl, and HNO_3_ were examined as back-extraction solvents. During this optimization study, other experimental conditions were adjusted to optimum values. Among all these solvents, HNO_3_ gave the superior extraction efficiency (>96%) and therefore, was chosen as the back-extraction solvent (Figure 3). After experiments, which were performed to explore the effects of HNO_3_ concentration and volume on the extraction efficiency, optimum HNO_3_ concentration and volume were found as 1 M and 500 *μ*L, respectively. (During these optimization experiments, concentration and volume of HNO_3_ were varied in the range of 0.1 M–2.0 M and 100 *μ*L–1000 *μ*L, respectively, and other parameters were kept at optimum values).

### 3.3. Effect of pH

**Figure 4:**
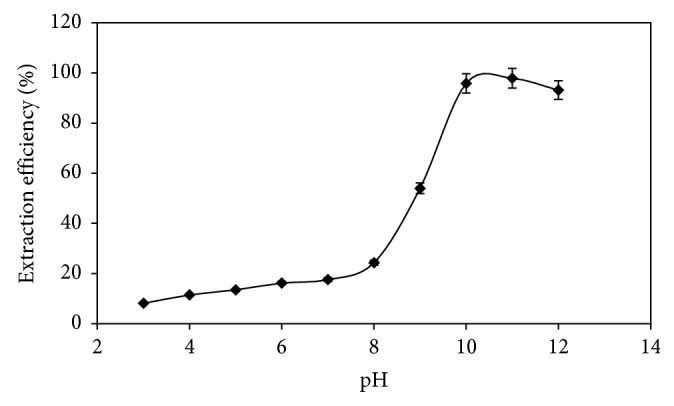
Effect of pH on the extraction efficiency. Conditions: 25 mL of 100 *μ*g·L^−1^ Pb^2+^, 0.5% (w/v) CR, 0.03% (v/v) DDMAC, 0.1 g NaCl, 75 *μ*L 1-dodecanol, 4 min extraction time, and 40°C extraction temperature.

Complex formation interaction between ligand and metal, and hence extraction efficiency, is pH-dependent [25, 26]. The effect of pH on the formation and extraction of Pb-CR complex was investigated at the pH range of 3.0–12.0 by adding appropriate buffer solutions. During these experiments, other parameters were adjusted to their optimum values. Obtained results are shown in Figure 4. As seen in this figure, the extraction efficiency of lead with CR into 1-dodecanol was maximized and remained nearly constant (>98%) in the pH range of 10.0–11.0. Decreasing extraction efficiencies at low pH values were probably due to the competition of hydrogen ions with lead ions in the complex formation reaction with CR. Therefore, the pH of aqueous solution was adjusted at around pH 10.0.

### 3.4. Effect of Amount of CR

The extraction efficiency of the analyte depends on the distribution ratio of the metal chelate between the organic and aqueous phases [27]. At a constant aqueous phase pH, the value of the distribution ratio and therefore the extraction efficiency increases to a certain value with the increasing amount of the chelate. The influence of CR amount on the extraction efficiency of lead was studied using different volumes of 0.1% (w/v) CR solution ranging from 0.1 mL to 2.0 mL, while other parameters were kept at their optimum values. According to the results obtained after optimization experiments, the extraction efficiency was increased with the increase of CR volume up to 0.5 mL and then remained constant at a value greater than 98%. In other words, the extraction efficiency was constant when the CR volume was higher than 0.5 mL, indicating complete complexation. Therefore, 0.5 mL of 0.1% (w/v) CR solution was chosen as the optimum value for subsequent studies.

### 3.5. Effect of DDMAC Concentration

**Figure 5:**
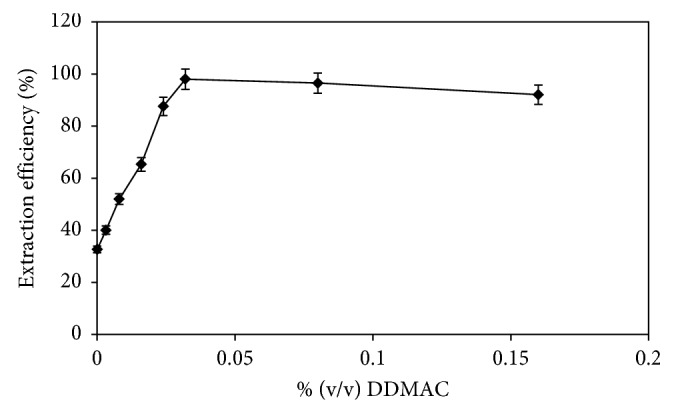
Effect of DDMAC concentration on the extraction efficiency. Conditions: 25 mL of 100 *μ*g·L^−1^ Pb^2+^, pH: 11.0, 0.5% (w/v) CR, 0.1 g NaCl, 75 *μ*L 1-dodecanol, 4 min extraction time, and 40°C extraction temperature.

In general, as mentioned before, species to be extracted into 1-dodecanol should have a hydrophobic character, and this is the greatest limitation of all the SFODME techniques. Consequently, hydrophilic and polar characters of the extracted compounds lower the extraction efficiency. To cope with this limitation, a cationic surfactant, DDMAC, has been introduced to the sample solution to attain sites with hydrophobic solubilizing properties that would make the extraction of metal chelates with hydrophilic character easier [28]. By adding DDMAC, the Pb-CR complex was possessed of more hydrophobic features, and thus, the complex was more extractable into 1-dodecanol. As a result, a higher extraction efficiency factor was obtained. In order to explore the effect of DDMAC concentration on the extraction efficiency, various amounts of DDMAC solution in the range of 0.003% to 0.16% were evaluated. The obtained results given in Figure 5 showed that with the increasing DDMAC concentration, the extraction efficiency increased up to 0.03% (v/v) and then remained constant up to 0.16% (v/v). So, a DDMAC concentration of 0.03% (v/v) was selected in the following experiments. Note that, the extraction efficiencies were about 30% and 98% in the absence and in the presence of DDMAC, respectively. So, it can be concluded that the presence of DDMAC enhances the extraction efficiency by more than 3 folds.

### 3.6. Effect of Salt Addition

Increase in ionic strength resulting from salt addition causes two opposite effects on the extraction efficiency: (1) the physical properties of the Nernst diffusion film can change by the presence of dissolved salt in water, and consequently, the diffusion rate of the analyte into the drop can decrease [29]; (2) the salting-out effect can cause an increase in the extraction efficiency [30]. In order to investigate the effect of ionic strength arising from salt addition, on performance of UA-DLLME-SFO-BE, various experiments were performed by adding varying amounts of NaCl from 0% to 4% (w/v). Other experimental parameters were kept constant. The results showed a gradual increase in the extraction efficiency of the Pb ions with increased NaCl amount up to 0.4% (w/v) and then remained constant when salt concentration is between 0.4% and 2%. However, a further increase in the salt concentration caused a decrease in the extraction efficiency. Thus, a concentration of 0.4% (w/v) of NaCl was chosen as the optimum value.

### 3.7. Effects of Temperature and Duration on Extraction and Back-Extraction

The temperature affects both the solubility of the organic extraction solvent in the aqueous phase and the emulsification process. Thus, it also affects the mass transfer and the extraction efficiency [28]. If the extraction temperature is lower than 25°C, the selected extraction solvent 1-dodecanol (melting point: 22–24°C) becomes more viscous in the extraction medium and the emulsification does not occur properly. On the contrary, at higher temperatures, the solubility and volatility of 1-dodecanol increase, and this leads to decrease in the extraction efficiency. In addition, higher temperatures cause decomposition of the metal complexes. In order to get a satisfying extraction efficiency, the extraction temperature and incubation time required to complete extraction should be optimized. The effect of the extraction temperature (ultrasonic bath temperature) and duration was explored in the range of 20–60°C and 1–8 min, respectively. It was found that a temperature of 40°C and a time of 4 min for sonication were adequate to achieve an optimum extraction efficiency. Same temperature and time values, 40°C and a 4 min, were used for back-extraction in UA-DLLME-SFO-BE.

### 3.8. Effect of Interfering Ions

Other ions except Pb in natural samples can affect the extraction efficiency. In order to explore the effect of coexisting ions, 25 mL solutions containing 50 *μ*g·L^−1^ Pb^2+^ ions together with foreign ions were processed according to the developed procedure. If an added foreign ion caused ±5% variation in the absorbance value of analyte, then it was considered as interfering species. The obtained results are summarized in Table 1. According to the given results, the method has a good tolerance to coexisting ions.

### 3.9. Analytical Performance of the Method

The calibration graphs were linear in the range of 10–500 *μ*g·L^−1^ lead under the optimum conditions of the proposed UA-DLLME-SFO-BE procedure. The regression equation for lead determination after microextraction was A = 4.22 × 10^−4^ C − 2.81 × 10^−2^, where A is the absorbance and C is the metal ion concentration in solution (*μ*g·L^−1^). The correlation coefficient of the calibration curve equation was 0.994. The equation obtained by direct aspiration in FAAS without the microextraction procedure was A = 5.22 × 10^−6^ C + 1.79 × 10^−4^, with linear range between 2,000 and 5,000 *μ*g·L^−1^ with the correlation coefficient of 0.999. The enhancement factor calculated as the ratio of the slope of calibration curves of the analyte after microextraction to that of prior microextraction was found as 81.

As an indication of precision, the relative standard deviation (RSD) was calculated for 10 replicate measurements by using 25 *μ*g·L^−1^ Pb^2+^ and was found to be less than 3.4%. The limit of detection (LOD), defined as the concentration ratio of three times the standard deviation of the blank signal and the slope of the calibration graph after preconcentration, was found as 1.9 *μ*g·L^−1^. The limit of quantification (LOQ) was the lowest level of the analyte that can be accurately and precisely measured. LOQ, defined as the concentration ratio of ten times the standard deviation of the blank signal and the slope of the calibration graph after preconcentration, was found as 6.4 *μ*g·L^−1^.  Table 2 summarizes some analytical figures of the method.

### 3.10. Accuracy of the Method

In order to prove the performance, the proposed method, a certified reference material (TM-61.2, fortified water), was used and recovery values were calculated. The certified concentration value for Pb(II) ions was 61.4 *μ*g·L^−1^. Recovery values were calculated as the averages of three parallel experiments and found as 99%. This value proves the satisfying consistency between the obtained results and certified value. These results also show that the developed method was successful for the determination of lead.

### 3.11. Analysis of Real Samples

In order to evaluate the validation of the method, the recovery experiments were performed by spiking different water samples such as tap (Ankara, Turkey) and lake (Ankara, Turkey) water samples. During these experiments, different amounts of lead were added to these water samples and the optimized method was applied. Obtained results are given in Table 3. As understood from the table, calculated recovery values for spiked water samples were always greater than 96%, and these results verify the validity of the proposed method.

## 4. Conclusion

An ultrasound-assisted dispersive liquid-liquid microextraction method based on solidification of floating organic drop and back-extraction (UA-DLLME-SFO-BE) technique for lead enrichment in aqueous samples prior to flame atomic absorption determination was reported in this paper. The novelty of the proposed method is the elimination of the clogging problem of the instrument parts caused by frozen 1-dodecanol. In addition, applying ultrasound energy shortens the extraction time and improves the extraction efficiency. Besides known advantages of the conventional SFODME method such as low cost, rapidity, simplicity of operation, high enhancement factor, and extraction efficiency, advantages originated from back-extraction make the proposed method very effective for the determination of trace amounts of lead in natural water samples.

## Figures and Tables

**Table 1:** Tolerance limits of interfering ions in the determination of 50 *μ*g·L^−1^ of Pb^2+^.

M^a^	[Pb^2+^]/[M]
Na^+^	>1 : 5000
Mg^2+^	>1 : 5000
Fe^3+^	1 : 500
Cd^2+^	1 : 1500
NH_4_^+^	>1 : 5000
Zn^2+^	1 : 500
Co^2+^	1 : 100
Cr^3+^	1 : 1000
Ni^2+^	1 : 100
SCN^−^	1 : 2000
Mn^2+^	1 : 500
CO_3_^2−^	1 : 1000
CrO_4_^2−^	1 : 2000
Cu^2+^	1 : 500
Al^3+^	1 : 100

^a^Interfering ion.

**Table 2:** Analytical characteristics of the proposed method.

Analytical feature	Value
Enhancement factor (EF)	81
Limit of detection (LOD) (*μ*g·L^−1^) (*n*=10)	1.9
Limit of quantitation (LOQ) (*μ*g·L^−1^) (*n*=10)	6.4
Linear range (*μ*g·L^−1^)	10–500
Precision (%RSD) (*n*=10, for 25 *μ*g·L^−1^ Pb^2+^)	3.4

**Table 3:** Determination of lead ions in several water samples (*n*=3).

Sample	Added (*μ*g·L^−1^)	Found (*μ*g·L^−1^)	Recovery %
Tap water	0	0	—
	25.0	24.1 ± 0.9	96.4
	50.0	48.6 ± 1.3	97.2
	100.0	98.1 ± 1.9	98.1

Lake water	0	7.6 ± 0.2	—
	25.0	31.9 ± 1.1	97.8
	50.0	56.1 ± 1.4	97.4
	100.0	106.2 ± 1.8	98.7

## Data Availability

Data obtained in this research are included in the article.
